# Lactobacillus acidophilus/Bifidobacterium infantis probiotics are associated with increased growth of VLBWI among those exposed to antibiotics

**DOI:** 10.1038/s41598-017-06161-8

**Published:** 2017-07-17

**Authors:** Christoph Härtel, Julia Pagel, Juliane Spiegler, Janne Buma, Philipp Henneke, Michael Zemlin, Dorothee Viemann, Christian Gille, Stephan Gehring, David Frommhold, Jan Rupp, Egbert Herting, Wolfgang Göpel

**Affiliations:** 1 Department of Pediatrics, University of Lübeck, Lubeck, Germany; 2 Center for Pediatrics and Adolescent Medicine and Center for Chronic, Immunodeficiency, University Medical Center, Freiburg, Germany; 3 Department of Pediatrics, University of Marburg, Marburg, Germany; 4 Department of Neonatology, Hanover Medical School, Hanover, Germany; 5 Department of Neonatology, University of Tübingen, Tubingen, Germany; 6 Department of Infectious Diseases, Gastroenterology and Pediatric Intensive Care, University of Mainz, Mainz, MD Germany; 7 Department of Neonatology, University of Heidelberg, Heidelberg, Germany; 8 Department of Infectious Diseases and Microbiology, University of Lübeck, Lubeck, Germany

## Abstract

We performed an observational study with very-low-birth weight infants (VLBWI) ≤33 weeks of gestation born in centers of the German Neonatal Network (GNN; (total n = 8534, n = 6229 received probiotics). The primary objectives of our study were (a) to assess the effect of *Lactobacillus acidophilus*/*Bifidobacterium infantis* probiotics on growth in VLBWI during primary stay in hospital and (b) to determine whether this effect is modified by antibiotic exposure. In linear regression models the administration of probiotics was independently associated with improved weight gain [g/d; effect size B = 0.62 (95% CI: 0.37–0.87), p < 0.001], and higher growth rates for body length [(mm/d; B = 0.06 (95% CI: 0.04–0.08), p < 0.001] and head circumference [mm/d; B = 0.03, 95% CI: 0.02–0.04, p < 0.001]. This effect was pronounced in infants with postnatal exposure to antibiotics; i.e. weight gain [g/d; B = 0.66 (95% CI: 0.32–1), p < 0.001], growth rate body length [(mm/d; B = 0.09 (95% CI: 0.06–0.12), p < 0.001] and head circumference [mm/d; B = 0.04, 95% CI: 0.02–0.06, p < 0.001]. In the small subgroup that was available for analysis at 5-year-follow-up (with probiotics: n = 120 vs. without probiotics: n = 54) we noted a sustained effect of probiotics in infants who received postnatal antibiotics. Probiotics may improve growth in antibiotic-treated infants which needs to be confirmed in randomized-controlled trials.

## Introduction

VLBWI are predisposed to early gut dysbiosis, which may increase the risk for acute, often fulminant complications such as sepsis or NEC. Gut dysbiosis may also lead to long lasting consequences, e.g. growth failure but also obesity and chronic inflammatory diseases^1–4^. Risk factors for gut dysbiosis in VLBWI include (a) prenatal administration of antibiotics to the mother leading to alteration of maternal microbiota composition, release of bacterial effectors and fetal antibiotic exposure, (b) Caesarean section, which prevents natural exposure to maternal bacteria, (c) perinatal infections, local or systemic inflammation and associated postnatal exposure to antibiotics. Probiotics may be a worthwhile treatment to foster the early microbiota establishment in a highly vulnerable population. They may have beneficial effects on growth, stabilization of the immunological homeostasis and thereby reduce the risk for infections and atopic disease^3, 5^. Studies on the therapeutic effects of probiotics in preterm infants have mainly focussed on short term endpoints, in particular NEC and sepsis. Several meta-analyses and systematic reviews including RCTs have concluded that prophylactic probiotics reduce the risk for NEC^6, 7^. In a large observational study in VLBW infants we have confirmed the association of *Lactobacillus acidophilus* and *Bifidobacterium infantis* probiotics with a reduced risk of NEC surgery^8^. This is in line with a recent analysis of the German NEO-KISS database indicating that the use of these dual-strain probiotics significantly reduced the risk of NEC^9^. Repa *et al*.^10^ reported the protective effect of *Lactobacillus acidophilus and Bifidobacterium infantis* on NEC in the subgroup of preterm infants exclusively fed with human milk, which was confirmed by a recent analysis from the Netherlands^11^. Despite these available data there is still uncertainty about the efficacy of probiotics. Recently, a large clinical trial involving 1315 infants ≤30 weeks of gestation found no clinical benefit of *Bifidobacterium breve* probotics for the risk of NEC^12^. The inconclusive results of well controlled trials have resulted in a very heterogenous incorporation of probiotics into clinical routine. While approximately 70% of VLBWI in Germany are prophylactically treated with probiotics^8^, most level III NICUs in the US are still reluctant to use probiotics as a clinical standard. It seems very likely that the inconsistency in probiotics efficacy is due to high variability in study protocols, including target populations, formulations – e.g. monostrain vs. multiple strains, and endpoints. In addition, the efficacy of probiotics could depend on the gut microbiota composition at baseline, i.e. before probiotics are started, which in turn depends on the history of antibiotic and pathogen exposure.

In order to improve strategies of prevention of dysbiosis and associated sequels including the use of probiotics, various endogenous and environmental influences need to be considered. This requires large, well-phenotyped cohorts, including subgroups with less exposure to antibiotics, i.e. VLBWI 28–32 weeks of gestation.. The primary objectives of our study were (a) to assess the effect of *Lactobacillus acidophilus*/*Bifidobacterium infantis* probiotics on growth in a large cohort of very- VLBWI (<33 weeks of gestation, n = 8534; subgroup 28–32 weeks, n = 5134) during primary stay in hospital and (b) to determine whether this effect is modified by ante- and postnatal antibiotic exposure.

## Results

### *Lactobacillus acidophilus*/*Bifidobacterium infantis* probiotics are associated with increased growth of VLBWI

#### Primary stay in hospital

The clinical characteristics of the cohort is outlined in Table 1 and supplemental Table 1. Mean duration of primary stay in hospital was 72 (median/25^th^–75^th^ percentile ± SD: 65/48–88 ± 34) days. In univariate analyses, VLBWI, who received probiotics (n = 6229) had a higher growth rate than infants without probiotics (n = 2305). To address whether velocity differences represent catch-up growth in children starting out smaller rather than effects of probiotics, we performed linear regression analyses including gestational age, birth weight, gender, multiple birth and maternal descent. As outlined in Table 2, probiotics were associated with improved weight gain [g/d; effect size B = 0.62 (95% CI: 0.37–0.87), p < 0.001], and higher growth rates for body length [(mm/d; B = 0.06 (95% CI: 0.04–0.08), p < 0.001] and head circumference [mm/d; effect size B = 0.03, 95% CI: 0.02–0.04, p < 0.001].

**Table 1:** Clinical characteristics according to prophylactic use of Lactobacillus acidophilus/Bifidobacterium infantis probiotics.

Clinical characteristics	All infants *without probiotics*	All infants *with probiotics*	p	28–32 weeks *without probiotics*	28–32 weeks *with probiotics*	p
No. of infants	2305	6229		1596	3538	
Gestational age (weeks)	29.0 (2.5)	28.3 (2.3)	<0.001*	30.3 (1.4)	30.0 (1.3)	<0.001*
Body weight at birth (g) z-score	1112 (297) −0.36/−0.36 (0.82)	1032 (291) −0.31/−0.25 (0.84)	<0.001* 0.002*	1251 (205) −0.44/−0.47 (0.74)	1203 (221) −0.42/−0.42 (0.77)	<0.001* 0.09*
Head circumference at birth (cm) z-score	26.1 (2.6) −0.41/−0.4 (0.77)	25.5 (2.5) −0.41/−0.37 (0.78)	<0.001* 0.4*	27.4 (1.6) −0.42/−0.42 (0.73)	27.0 (1.6) −0.42/−0.37 (0.75)	<0.001* 0.9*
Body length at birth (cm) z-score	37.1 (3.8) −0.26/−0.22 (0.8)	36.2 (3.7) −0.23/−0.18 (0.81)	<0.001* 0.08*	38.8 (2.6) −0.32/−0.29 (0.78)	38.2 (2.8) −0.33/−0.24 (0.81)	<0.001* 0.6*
SGA	12.7	12.1	0.4	11.4	11.9	0.7
Female gender	48.7	48.9	0.9	50.5	49.3	0.4
Multiple birth	34.8	35.2	0.7	37	38.3	0.4
Caesarean section	90.9	91.3	0.9	92	92.3	0.7
Duration of stay (days)	7035	7334	<0.001*	5319	5519	0.009*
Weight gain (g/d)	23.1 (6.0)	23.1 (5.6)	0.4*	24.5 (5.9)	24.3 (5.8)	0.05*
Growth velocity head (mm/d)	1.02 (0.3)	1.05 (0.3)	0.01*	1.04 (0.3)	1.06 (0.3)	0.3*
Growth velocity length (mm/d)	1.37 (0.5)	1.43 (0.5)	<0.001*	1.39 (0.4)	1.43 (0.4)	0.007*

Legend: Continuous variables are shown as mean (SD); z-scores are shown as mean/median (SD). Categorical variables are shown as percent. p-values were derived from Fisher’s exact test or Mann-Whitney-U test if indicated (*), SGA, small for gestational age (<10th percentile of birth weight adjusted to gestational age). Growth velocity and weight gain were calculated by differences between parameters at birth and respective measures at discharge/number of days (duration of stay).

**Table 2:** Effect of probiotics on growth during primary stay in hospital.

Variable	All infants (n = 8516)	28–32 weeks (n = 5127)
**Weight gain** (**g/d)**		
Effect of Probiotics	0.62	−0.05
95% CI; p	0.37–0.87; <0.001	−0.29–0.39; 0.8
Adjusted R^2^	0.12	0.03
**Growth velocity body length** (**mm/d)**		
Effect of Probiotics	0.06	0.03
95% CI; p	0.04–0.08; <0.001	−0.002–0.006; 0.06
Adjusted R^2^	0.01	0.007
**Growth velocity head circumference** (**mm/d)**		
Effect of Probiotics	0.03	0.006
95% CI	0.02–0.04; <0.001	−0.01–0.02; 0.5
Adjusted R^2^	0.02	0.02

Linear regression analysis included gestational age per week, birth weight in 100 g steps, gender, multiple birth, maternal descent and exposure to *Bifidobacterium infantis*/*Lactobacillus acidophilus* probiotics. Linear regression analysis included gestational age per week, birth weight in 100 g steps, gender, multiple birth, maternal descent and exposure to *Bifidobacterium infantis*/*Lactobacillus acidophilus* probiotics. The effect of probiotics on weight gain as g/d growth rates in mm/d is shown as B coefficient, 95% confidence interval and p-value. Adjusted R^2^ values indicate coefficients of determinations and adjust for the number of terms used in the model.

#### Subgroup of VLBWI born 28 0/7– 32 6/7 weeks

In the subgroup of infants ≥28 0/7 ≤ 32 6/7 gestational weeks probiotics significantly accelerated growth rate for body length (1.43 vs. 1.39 mm/d, p = 0.007; adjusted effect size B = 0.03, p = 0.06), while other growth parameters were not affected by probiotic use (Tables 1 and 2).

#### 2-year-follow up

Based on the information retrieved from the parents with the standardized questionnaires^13^, infants who received probiotics in the early neonatal period (n = 813) were not different from infants without probiotics (n = 333) with regard to number of upper respiratory tract infections in the first 24 months of life or risk for atopic dermatitis. In VLBWI born 28–32 weeks of gestation (n = 724), we noted a potential risk reduction for the mean ( ± SD) number of episodes for upper airway infections in the first 24 months after discharge from primary stay in hospital (2.8 ± 2.4 vs. 3.2 ± 2.3, p = 0.02; Table 3).

**Table 3:** Follow-up data according to prophylactic use of Lactobacillus acidophilus/Bifidobacterium infantis probiotics.

Clinical characteristics	All infants *Without probiotics*	All infants *with probiotics*	p	28–32 weeks *without probiotics*	28–32 weeks *with probiotics*	p
No. of infants	54	120		23	46	
Gestational age (weeks)	27.5 (2.3)	27.6 (2.3)	0.9*	29.7 (1.3)	30.0 (1.4)	0.5*
Body weight (g) at birth	940290	964297	0.1*	1157251	1202278	0.1*
Body weight (kg) at 5yr-F/U z-score	16.9 (2.4) −0.93/−0.91 (1.03)	18.0 (3.5) −0.43/−0.63 (1.4)	0.08^*^ 0.06*	17.1 (2.9) −0.89/−1.19 (1.23)	18.5 (3.1) −0.21/−0.52 (1.22)	0.1^*^ 0.06*
Head circumference (cm) at 5 yr F/U; z-score	49.5 (2.1) −1.39 (−1.33, 1.73)	50.2 (1.8) −0.71 (−0.0, 1.35)	0.009^*^ 0.006*	50.1 (2.2) −0.94 (−1, 1.67)	50.3 (1.6) −0.56/−0.69 (1.15)	0.2^*^ 0.2*
Body length (cm) at 5 yr F/U z-score	108.7 (5.2) −0.56/−0.60 (1.08)	110.3 (5.6) −0.21/−0.35 (1.13)	0.09^*^ 0.2*	107.9 (6.1) −0.83/−0.51 (1.17)	110.8 (5.8) −0.06/−0.35 (1.13)	0.09_*_ 0.05*
Episodes with upper respiratory tract infection at 2 yr F/U no. of infants [mean (SD) GA, weeks)] mean number of episodes (SD)	333 [28.9 (2.5)] 3.0 (2.3)	819 [28.3 (2.3)] 2.8 (2.3)	0.1*	228 [28.9 (2.5)] 3.2 (2.3)	496 [28.9 (2.5)] 2.8 (2.4)	0.02*
Atopic dermatitis at 2 yr F/U (%)	10.3	13.5	0.3	9.8	12.7	0.3

Legend: Continuous variables are shown as mean (SD); z-scores are shown as mean/median (SD). Categorical variables are shown as percent. p-values were derived from Fisher’s exact test or Mann-Whitney-U test if indicated (*), SGA, small for gestational age (<10th percentile of birth weight adjusted to gestational age). Growth velocity and weight gain were calculated by differences between parameters at birth and respective measures at discharge/number of days (duration of stay).

#### 5-year-follow-up

In the small subgroup that was available for analysis at 5-year-follow-up (with probiotics: n = 120 vs. without probiotics: n = 54) we noted a sustained effect of probiotics on growth for head circumference (Table 3) which was independent from gestational age, birth weight, gender, multiple birth and maternal descent (cm; effect size B = 0.77, 95% CI: 0.16–1.38, p = 0.01).

### Modification of the effect of probiotics on growth by antibiotic exposure

#### Antibiotic exposure

**Figure 1:**
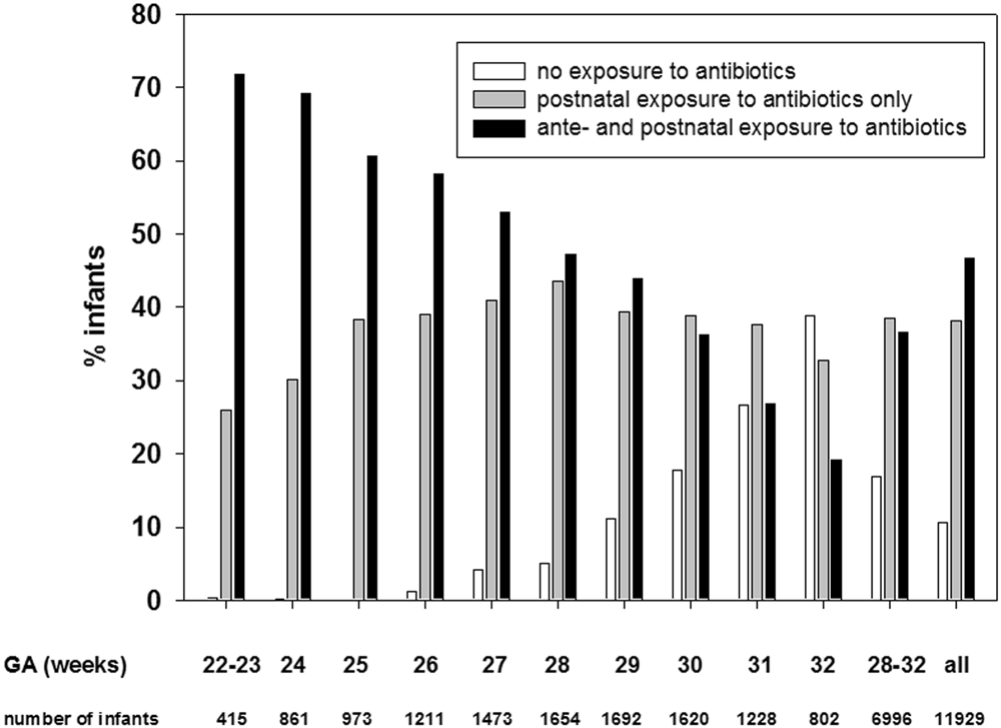
Exposure to antenatal and postnatal antibiotics according to gestational age. The figure describes the percentage of infants exposed to antenatal and/or postnatal antibiotics. A small percentage of infants was exposed to antenatal antibiotics but not to postnatal antibiotics (22–23 weeks: 1.4%, 24 weeks: 0.5%, 25 weeks: 0.8%; 26 weeks: 1.4%, 27 weeks: 2.0%; 28 weeks: 4.1%, 29 weeks: 5.6%; 30 weeks: 7.3%, 31 weeks: 9.4%; 32 weeks: 9.2%, 28–32 weeks: 6.6%; all: 4.5%).

As depicted in Fig. 1, exposure to antibiotics is high across all gestational ages. Specifically, only 10.7% were never exposed to antenatal antibiotics or treated with postnatal antibiotics (stratum 1), 4.5% were exposed to antenatal antibiotics only (stratum 2), 38.2% had direct administration of postnatal antibiotics only (stratum 3), and 46.7% had exposure to both, ante- and postnatal antibiotics (stratum 4). Even in lower risk VLBWI, i.e. those with a gestational age of ≥28 0/7 ≤ 32 6/7 weeks, the percentage of infants who were not exposed to antibiotics was low (16.8%). In this subgroup, 6.6% had antenatal exposure only, 38.5% had postnatal treatment only and 36.5% were exposed to antibiotics before and after birth.

#### Antibiotic classes

51.2% of mothers of VLBWI were exposed to antibiotics less than 5 days before preterm birth. Antenatal antibiotics were administered for several reasons, with preterm labour and suspected chorioamnionitis being the predominant causes. Cephalosporins and penicillins were used most frequently by the obstetricians (Supplemental Table 2). Neonatologist administered penicillins and aminoglycosides most often to VLBWI, while a significant number of infants was also exposed to second or third line antibiotics, such as glycopeptides (i.e. vancomycin) or carbapenems (Supplemental Table 3).

Within the four strata of antibiotic exposure, the percentage of infants supplemented with *Lactobacillus acidophilus*/*Bifidobacterium infantis* probiotics was different (68% without antenatal/postnatal exposure, 74% with antenatal exposure, 71% with postnatal exposure and 76.3% with both antenatal and postnatal exposure, p < 0.001).

### Probiotic effect and modification by antibiotics

#### Antenatal antibiotics

Probiotics did not show a growth promoting effect in VLBWI, who were not exposed to antibiotics (stratum 1), or in VLBWI, who were exposed to antenatal antibiotics only (stratum 2, Table 4).

**Table 4:** Antenatal antibiotics and effect of probiotics.

Clinical characteristics	No antibiotics *without probiotics*	No antibiotics *with probiotics*	p	Antenatal antibiotics only *without probiotics*	Antenatal antibiotics only *with probiotics*	p
No. of infants	295	628		103	293	
Gestational age (weeks)	31.0 (1.3)	30.4 (1.5)	<0.001	30.7 (1.4)	29.8 (1.7)	<0.001
Body weight at birth (g) z-score	1277 (190) −0.7/−0.77 (0.67)	1189 (221) −0.67/−0.74 (0.74)	<0.001 0.7	1300 (175) −0.49/−0.53, (0.67)	1228 (224) −0.3/−0.28, (0.76)	0.001 0.02
Head circum-ference at birth (cm) z-score	27.8 (1.5) −0.5/−0.47 (0.69)	27.1 (1.7) −0.55/−0.49, (0.75)	<0.001 0.6	27.8 (1.4) −0.36/−0.37, (0.61)	27.0 (1.7) −0.36/−0.29, (0.75)	<0.001 0.8
Body length at birth (cm) z-score	39.3 (3.4) −0.48/−0.48, (0.74)	38.2 (2.7) −0.51/−0.5, (0.79)	<0.001 0.4	39.3 (2.2) −0.32/−0.24, (0.71)	38.3 (2.8) −0.22/−0.21, (0.72)	0.001 0.3
Duration of stay (days)	45 (13)	49 (16)	0.001	45 (13)	49 (15)	0.07
Weight gain (g/d)	26.2 (6.6)	24.9 (57.1)	0.01	26.1 (4.3)	25.3 (14)	0.001
Growth velocity head (mm/d)	1.10 (0.3)	1.09 (0.3)	0.8	1.08 (0.3)	1.09 (0.3)	0.8
Growth velocity length (mm/d)	1.49 (0.6)	1.45 (0.5)	0.3	1.34 (0.5)	1.46 (0.6)	0.08

Legend: Continuous variables are shown as mean (SD); z-scores are shown as mean/median (SD). Categorical variables are shown as percent. p-values were derived from Fisher’s exact test or Mann-Whitney-U test if indicated (*), SGA, small for gestational age (<10th percentile of birth weight adjusted to gestational age). Growth velocity and weight gain were calculated by differences between parameters at birth and respective measures at discharge/number of days (duration of stay).

#### Postnatal antibiotics

**Figure 2:**
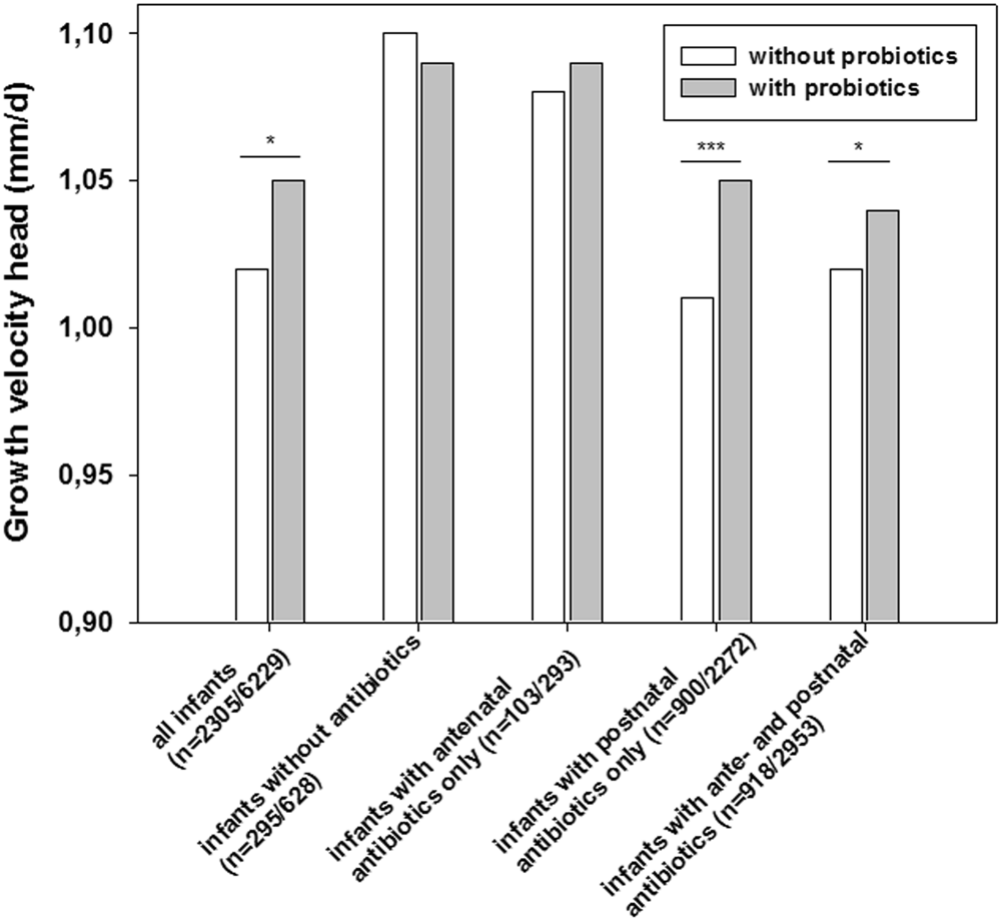
The effect of probiotics and modification by antibiotics on head circumference. The figure describes the effect of probiotics on growth velocity [difference of birth and discharge measures/number of days (duration of primary stay in hospital)] for head circumference. The effect is modified by exposure to antibiotics, the different strata are depicted on the x-axis. For each stratum, numbers of infants without probiotic treatment/with probiotic treatment are given. *p < 0.05, **p < 0.01, ***p < 0.001 (Fisher’s exact test, two-sided).

**Figure 3:**
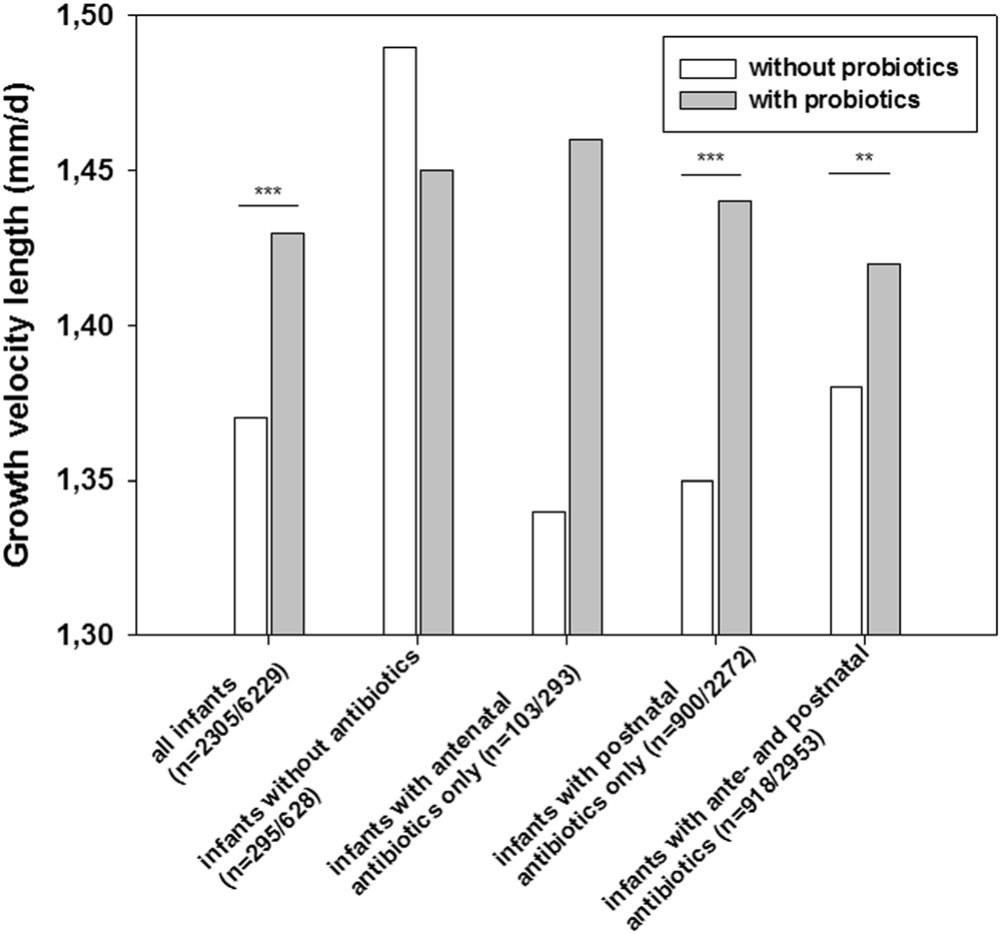
The effect of probiotics and modification by antibiotics on body length. The figure describes the effect of probiotics on growth velocity [difference of birth and discharge measures/number of days (duration of primary stay in hospital)] for body length. The effect is modified by exposure to antibiotics, the different strata are depicted on the x-axis. For each stratum, numbers of infants without probiotic treatment/with probiotic treatment are given. *p < 0.05, **p < 0.01, ***p < 0.001 (Fisher’s exact test, two-sided).

Probiotics accelerated growth in VLBWI with postnatal antibiotic exposure [stratum 3; with probiotics: n = 2272 vs. without probiotics: n = 900; mean HC ± SD (mm/d): 1.05 ± 0.3 vs. 1.01 ± 0.3, p < 0.001; mean BL ± SD (mm/d): 1.44 ± 0.4 vs. 1.35 ± 0.4, p < 0.001] and stratum 4 (Table 5; Fig. 2). As described in Table 6, probiotics were associated with improved growth rate in stratum 3 infants; i.e. weight gain [g/d; B = 0.66 (95% CI: 0.32–1), p < 0.001], growth rate body length [(mm/d; B = 0.09 (95% CI: 0.06–0.12), p < 0.001] and head circumference [mm/d; B = 0.04, 95% CI: 0.02–0.06, p < 0.001] and in stratum 4 infants (Figs 2 and 3).

**Table 5:** Postnatal antibiotics and effect probiotics.

Clinical characteristics	Postnatal antibiotics only *without probiotics*	Postnatal antibiotics only *with probiotics*	p	Ante - and postnatal antibiotics *without probiotics*	Ante - and postnatal antibiotics *with probiotics*	p
No. of infants	900	2272		918	2953	
Gestational age (weeks)	29.0 (2.4)	28.4 (2.2)	<0.001	28.2 (2.5)	27.6 (2.3)	<0.001
Body weight at birth (g) z-score	1070 (306) −0.53/−0.57 (0.85)	988 (293) −0.52/−0.5, (0.89)	<0.001 0.7	1090 (299) −0.07/−0.04, (0.74)	1014 (290) −0.06/−0.02, (0.75)	<0.001 0.7
Head circumference at birth (cm) z-score	26.0 (2.6) −0.52/−0.5, (0.79)	25.4 (2.4) −0.54/−0.5, (0.8)	<0.001 0.7	25.7 (2.6) −0.29/−0.26, (0.76)	25.1 (2.6) −0.28/−0.25, (0.74)	<0.001 0.6
Body length at birth (cm) z-score	36.7 (3.9) −0.38/−0.33, (0.85)	35.7 (3.8) −0.4/−0.33, (0.87)	<0.001 0.9	36.6 (3.9) −0.07/0 (0.73)	35.8 (3.7) −0.04/−0.03, (0.72)	<0.001 0.4
Duration of stay (days)	72 (33)	75 (33)	<0.001	77 (38)	79 (35)	0.003
Weight gain (g/d),	22.7 (5.4)	22.9 (4.5)	0.9	22.7 (5.9)	22.8 (4.4)	0.7
Growth velocity head (mm/d)	1.01 (0.2)	1.05 (0.3)	<0.001	1.02 (0.3)	1.04 (0.3)	0.03
Growth velocity length (mm/d)	1.35 (0.4)	1.44 (0.4)	<0.001	1.38 (0.5)	1.42 (0.4)	0.001

Legend: Continuous variables are shown as mean (SD); z-scores are shown as mean/median (SD). Categorical variables are shown as percent. p-values were derived from Fisher’s exact test or Mann-Whitney-U test if indicated (*), SGA, small for gestational age (<10th percentile of birth weight adjusted to gestational age). Growth velocity and weight gain were calculated by differences between parameters at birth and respective measures at discharge/number of days (duration of stay).

**Table 6:** Effect of probiotics on growth during primary stay in hospital.

Variable	No antibiotics (n = 919)	Antenatal antibiotics only (n = 396)	Postnatal antibiotics only (n = 3166)	Ante- and postnatal antibiotics (n = 3862)
**Weight gain** (**g/d)**				
Effect of Probiotics	−0.6	−0.06	0.66	0.48
95% CI; p	−1.29 −0.15; 0.1	−2.8–2.7; 0.9	0.32–1; p < 0.001	0.14–0.8; 0.005
Adjusted R^2^	0.07	0.004	0.14	0.12
**Growth velocity body length** (**mm/d)**				
Effect of Probiotics	−0.05	0.1	0.09	0.03
95% CI; p	−0.12–0.03; p = 0.2	−0.04–0.24; 0.2	0.06–0.12; p < 0.001	0–0.06; 0.05
Adjusted R^2^	0.001	0.009	0.012	0.004
**Growth velocity head circumference** (**mm/d)**				
Effect of Probiotics	−0.02	0.014	0.04	0.02
95% CI;p	−0.07–0.02; 0.3	−0.06–0.09; 0.7	0.02–0.06; p < 0.001	0.004–0.04; 0.02
Adjusted R^2^	0.03	0.003	0.02	0.01

Linear regression analysis included gestational age per week, birth weight in 100 g steps, gender, multiple birth, maternal descent and exposure to *Bifidobacterium infantis*/*Lactobacillus acidophilus* probiotics. Linear regression analysis included gestational age per week, birth weight in 100 g steps, gender, multiple birth, maternal descent and exposure to *Bifidobacterium infantis*/*Lactobacillus acidophilus* probiotics. The effect of probiotics on weight gain as g/d growth rates in mm/d is shown as B coefficient, 95% confidence interval and p-value. Adjusted R^2^ values indicate coefficients of determinations and adjust for the number of terms used in the model.

At 2 year follow-up, stratum 3 infants with postnatal antibiotic exposure only seem to have a benefit from neonatal probiotic use with regard to a potential risk reduction for the mean ( ± SD) number of episodes for upper airway infections (2.6 ± 2.1 vs. 3.1 ± 2.6, p = 0.01; Table 7) during the first 24 months after discharge from hospital. The risk for atopic dermatitis was not affected. All other strata of antibiotic exposure did not benefit from probiotics.

**Table 7:** Follow-up data of infants exposed to postnatal antibiotics according to prophylactic use of Lactobacillus acidophilus/Bifidobacterium infantis probiotics.

Clinical characteristics	Postnatal exposure to antibiotics *Without probiotics*	Postnatal exposure to antibiotics *with probiotics*	p	Ante- and postnatal exposure to antibiotics *without probiotics*	Ante- and postnatal exposure to antibiotics *with probiotics*	p
No. of infants	19	46		29	61	
Gestational age (weeks)	27.2 (2.3)	27.9 (2.1)	0.9*	27.2 (2.1)	26.9 (2.1)	0.8*
Body weight (g) at birth	787 (258)	926 (272)	<0.001*	976 (262)	928 (290)	0.1*
Body weight (kg) at 5yr-F/U z-score	15.8 (2.0); −1.46/−1.62 (0.84)	18.2 (4.2) −0.31/−0.67 (1.74)	0.008* 0.003*	17.6 (2.4) −0.54/−0.66 (1.01)	17.8 (3.1) −0.55/−0.59 (1.3)	1.0* 0.9*
Head circumference (cm) at 5 yr F/U, z-score	48.9 (1.4) 1.68/−1.75 (1.19)	49.9 (1.8) −0.83/−0.54 (1.32)	0.01* 0.01*	49.9 (2.5) −1.32/−1.04 (2.07)	50.4 (1.9) −0.72/−0.77 (1.42)	0.3* 0.3*
Body length (cm) at 5 yr F/U z-score	107.0 (6.0) −0.91/−1.1 (1.27)	110.0 (6.0) −0.2/−0.71 (1.25)	0.06* 0.07*	109.7 (4.3) −0.31/−0.11 (0.83)	110.1 (5.3) −0.23/−0.21 (1.06)	0.8* 0.9*
Episodes with upper respiratory tract infection at 2 yr F/U, no. of infants [mean (SD) GA, weeks)] mean number of episodes (SD)	134 [28.7 (2.5)] 3.1 (2.6)	318 [28.4 (2.3)] 2.6 (2.1)	0.01*	138 [28.2 (2.5)] 2.8 (2.3)	406 [27.6 (2.3)] 2.8 (2.3)	0.8*
Atopic dermatitis at 2 yr F/U (%)	9.2	13.6	0.1	12.7	13.8	0.3

Legend: Continuous variables are shown as mean (SD); z-scores are shown as mean/median (SD). Categorical variables are shown as percent. p-values were derived from Fisher’s exact test or Mann-Whitney-U test if indicated (*), SGA, small for gestational age (<10th percentile of birth weight adjusted to gestational age). Growth velocity and weight gain were calculated by differences between parameters at birth and respective measures at discharge/number of days (duration of stay).

At 5-year-follow-up, the effect of probiotics on weight gain/growth was solely persistant in stratum 3 infants [with probiotics: n = 46; without probiotics: n = 19; mean body weight (BW) ± SD (kg): 18.2 ± 4.2 vs. 15.8 ± 2.0, p = 0.008; mean HC ± SD (cm): 49.9 ± 1.8 vs. 48.9 ± 1.8, p = 0.01; mean BL ± SD (cm): 110.0 ± 6.0 vs. 107.0 ± 6.0, p = 0.06, Table 6]. This was confirmed after adjustment for confounding variables (Table 8).

**Table 8:** Effect of probiotics on growth parameters at 5 years of age.

Variable	all (n = 151)	Postnatal antibiotics only (n = 59)	Ante- and postnatal antibiotics (n = 74)
**Body weight**			
Effect of Probiotics	1.2	3.3	0.1
95% CI; p	0.1–2.3; 0.04	1.13–5.43; 0.003	−1.7–1.5; 0.9
Adjusted R^2^	0.06	0.16	0.11
**Body length** (**cm)**			
Effect of Probiotics	1.59	4.4	0.4
95% CI; p	−0.3–3.4; 0.09	0.97–7.8; 0.01	−2–2.8; 0.7
Adjusted R^2^	0.1	0.2	0.15
**Head circumference** (**cm)**			
Effect of Probiotics	0.77	0.91	0.7
95% CI; p	0.16–1.38; 0.01 0.23	0.43–1.79; 0.04	−0.3–1.7; 0.2
Adjusted R^2^		0.29	0.22

Linear regression analysis included gestational age per week, birth weight in 100 g steps, gender, multiple birth, maternal descent and exposure to *Bifidobacterium infantis*/*Lactobacillus acidophilus* probiotics. Linear regression analysis included gestational age per week, birth weight in 100 g steps, gender, multiple birth, maternal descent and exposure to *Bifidobacterium infantis*/*Lactobacillus acidophilus* probiotics. The effect of probiotics on weight gain as g/d growth rates in mm/d is shown as B coefficient, 95% confidence interval and p-value. Adjusted R^2^ values indicate coefficients of determinations and adjust for the number of terms used in the model.

## Discussion

In a large population-based cohort study we demonstrated that supplementation with *Lactobacillus acidophilus*/*Bifidobacterium infantis* probiotics improved growth of VLBWI during primary stay in hospital. This effect is most pronounced in infants with postnatal exposure to antibiotics. Given the limitations of an epidemiological analysis our data are hypothesis-generating. Administration of dual-strain probiotics may improve the metabolism of VLBWI and therefore considered as medical intervention that can target the microbiota-host interplay at the beginning of life, with potentially long-lasting impact on individual health.

Microbial patterns of initial colonisation of the intestine are important for growth of the newborn as they support the gut integrity, nutrient absorption as well as metabolic and endocrine functions (fat deposition, leptin and insulin levels; ref. 14. Bioactive compounds are known to stimulate the immune system, to support the infant’s growth and to facilitate the selective colonization of apparently protective bacterial species such as *Bifidobacteria* and *Lactobacilli* ^15, 16^.

Preterm birth, however, is associated with particular challenges to the development of the delicate host-microbe mutualism including mode of delivery, immature immunity, exposure to antibotics as well as feeding strategies. In the complex situation of preterm infants, probiotics may have a positive effect on infant´s growth. This assumption is based on animal models that reported improved weight gain in poultry that received probiotic supplement^17^. The beneficial effects of probiotics on metabolism may be mediated by the production of enzymes for fermentation of non-digestible dietary residues, energy recovery in the form of short-chain fatty acids, absorption of electrolytes and iron, synthesis of vitamins and conversion of pro-drugs to active metabolites. Furthermore, probiotics may help to repress potentially virulent bacteria by competition for compounds^18^. It is important to note, that the host dictates many of the conditions under which different bacteria of the microbiota compete. Thus it remains an important research question how the host’s genotype and the environmental factors interact to establish eubiosis or dysbiosis in the gut.

Clinical trials on the effect of probiotics on neonatal growth parameters are scarce. Term infants who are not nourished with human milk might have improved growth when formula is supplemented with Bifidobacterium lactis^19^. In preterm infants observational studies suggested a potential relationship between the diversity of the intestinal microbiota and weight gain in VLBWI^20^. A small scale study from Japan including 91 VLBWI suggested that supplementation with Bifidobacterium breve might improve gastrointestinal tolerance and weight gain^21^. So far, randomised trials which studied weight gain as main outcome failed to demonstrate a beneficial effect of probiotics^22^. Just recently, the PREMAPRO study performed by Hays *et al*.^23^ randomly assigned 199 preterm infants to receive daily supplementation over 4–6 weeks with placebo or three groups receiving probiotics - Bifidobacterium lactis or Bifidobacterium longum, or both. At the end of the supplementation period, no significant differences were seen for body weight, length, and head circumference.

Our large scale data point to a specific role of probiotics in the context of antibiotic exposure. In our setting, >85% infants are exposed to ante- and/or postnatal antibiotics. Even in the subgroup of infants with lower risk (28–32 weeks of gestation) treatment rate was >75%. Interestingly, infants who were not exposed to antibiotics or only exposed to antenatal antibiotics did not benefit from supplementation with probiotics.

Our primary observation is that antibiotic-treated VLBWI benefit from probiotics. In mature, established microbiota antibiotic treatment is unlikely to result in persistent changes^24^. In contrast, the developing infant gut microbiota of preterm infants is highly dynamic and susceptible to disruption by antibiotic exposure^4, 25^. For example, macrolide use in 2–7 year-old Finnish children is associated with a long-lasting shift in microbiota composition and metabolism^26^. Antibiotic treatment of newborns causes a reduced prevalence of *Clostridia*, whereas the gut of untreated infants was more likely to be colonized with *Escherichia coli* and *S*. *aureus* ^27^. Gibson *et al*. recently^28^ noted that antibiotic treatments in VLBWI are associated with widespread collateral microbiome impact by enrichment of antibiotic resistance genes that have no known activity against the specific antibiotic driver. With high-resolution microbiota sequencing, the gut dysbiosis in preterm infants mediated by antibiotics may be characterized by four aspects: loss of keystone taxa, loss of diversity, shifts in metabolic capacity, and blooms of pathogens^4^. We hypothesize that probiotics may prevent or attenuate the adverse effects of antibiotics on gut communities thereby stabilizing gut integrity and improving absorption of nutrients. This effect might be sustainable on weight, body length and head circumference at the age of 5 years, at least in the subgroup of infants who were treated with postnatal antibiotics. This aspect would be highly beneficial to preterm infants who are prone to growth failure as compared to term infants; i.e. the antibiotic-treated subgroup of infants who benefitted most from probiotic supplementation had still impaired growth at 5 years of age; mean weight: 18 kg = 2 kg <50^th^ percentile, mean body length 110 cm = 3 cm <50^th^ percentile, mean head circumference: 50 cm = 1 cm <50^th^ percentile as compared to KiGGS data; 13). A single small RCT on the effect of Bifidobacterium lactis Bb12 demonstrated that in antibiotic-treated infants, probiotic supplementation resulted in a higher body weight, lower fecal pH, lower fecal calprotectin and higher fecal IgA levels as compared with placebo^29^, therefore supporting our finding of improved growth parameters in antibiotic-treated infants supplemented with probiotics. Secondly, we hypothesize that probiotics prevent low-grade chronic inflammation^3, 5^. This chronic inflammation is usually associated with higher energy consumption and predisposing to growth failure. On the other hand, probiotics may have beneficial effects on the crosstalk between metabolism and developing immune system which is is not yet understood. Antibiotic-treated infants in our setting have a lower rate of upper respiratory tract infections during infancy when supplemented with probiotics after birth. This needs to be confirmed in large-scale studies.

Our approach has several limitations. We present data of an observational study which may be biased by several confounding variables including center-specific effects and uneven number of VLBWI who were not treated with probiotics. In addition to that, the subgroup of infants with follow-up data at 5 years is small yet (as GNN has started enrolment in 2009 with available data on probiotic use in 2010). The sample size of the follow-up cohort is not sufficient to draw warrant conclusions. Secondly, probiotics were more often given to infants <28 weeks of age. A part of the effects on growth may be more related to catch-up growth of extremely preterm infants rather than a pure probiotic effect. This may explain why duration of stay in hospital might be shorter for infants without probiotics, despite enhanced weight gain in the probiotics group. Thirdly, the administration of probiotics for 28 days in capsules is a pragmatical way but still arbitrary and variable among study centers. Finally, duration of primary stay in hospital was highly variable (median 65 days, 25^th^–75^th^ percentile: 48–88 days) which implies variability in observational time and outcome measures for growth. In conclusion, large randomized-controlled trials and animal models are needed to clarify the efficacy and mechanism of probiotics for preventing long-term health problems of preterm infants, i.e. infections and growth failure. Furthermore, the effect of probiotics may vary depending on the species and the strains/mix of microorganisms employed as well as the feeding strategies (human milk vs. formula) used in different centers. Deep sequencing technology may enable to discover new probiotic formulations to guide preventive strategies against dysbiosis^30^.

## Methods

### Observational study

The German Neonatal Network (GNN) studies the long-term effects of genetic, clinical, and social risk factors as well as center specific treatment strategies in very-low-birth weight infants (VLBWI) born in 54 neonatal intensive care units in Germany (1^st^ of April 2009 until 31^st^ of December 2015, n = 11929 enrolled infants). Herein we performed an observational, population-based study with VLBWI enrolled in the first days after birth. The inclusion criteria for this study were as follows: birth weight <1500 g and gestational age >22 0/7 and ≤32 6/7 weeks, written informed consent of pareets or legal representatives and discharge to home environment Exclusion criteria were lethal malformations, e.g. trisomy 13 and trisomy 18. After recruitment by the attending physicians, a predefined GNN data set (supplemental information), including treatment parameter and outcome data, was recorded by completion of case record files. After discharge, data sheets were sent to the GNN center in Lübeck. A physician trained in neonatology or a study nurse evaluated the data quality by annual on site monitoring of the data sets. Data on the use of *Lactobacillus acidophilus*/*Bifidobacterium infantis* (Infloran®, one capsule containing 10^9^ *L*. *acidophilus* and 10^9^ *B*. *infantis*) probiotics were documented from 1^st^ of September 2010 until 31^st^ of December 2015 (n = 8534) at 48 study sites. We included subgroup analyses as follows:

VLBWI born at 28 0/7–32 6/7 gestational weeks (n = 5134)

All VLBWI without exposure to antibiotics (stratum 1, n = 923)

All VLBWI with exposure to antenatal antibiotics only (stratum 2, n = 396)

All VLBWI with postnatal antibiotic treatment only (stratum 3, n = 3172)

All VLBWI with antenatal and postnatal antibiotic treatment (stratum 4, n = 3871).

The primary objectives of our study were (a) to assess the effect of prophylactic *Lactobacillus acidophilus*/*Bifidobacterium infantis* probiotics on growth in very-low-birth-weight infants (VLBWI) during primary stay in hospital and (b) to determine whether this effect is modified by ante- and postnatal antibiotic exposure. This study was not nested within a clinical trial.

### Follow-up analysis

#### 24-month-follow-up

For the 24-month-follow-up, parents of surviving infants enrolled in GNN received a voluntary questionnaire (according to the German Health Interview and Examination Survey for Children and Adolescents (KiGGS) from Robert Koch Institute, Germany including data on sociodemographic characteristics, vaccine preventable diseases, and illnesses such as infections and atopic disease; www.rki.de)^13^. Prevalence calculations of infections were based on the question: “Has your child ever had the following infectious illnesses…?” Possible parental responses were “yes”/”no”/”don’t know”. The parent questionnaire collected data on the following infections after discharge from primary stay in hospital: cold/flu-like infection, tonsillitis, herpesvirus infection, bronchitis (not when asthma was present), gastrointestinal infection, cystitis and/or urethritis, purulent conjunctivitis (bacterial conjunctivitis).

#### 5-year-follow-up

For the 5-year follow-up infants were examined by the GNN study team (physician trained in neonatology and 2 study nurses). Growth parameters (body length, body weight, head circumference) and systolic/diastolic blood pressure levels were determined with standardized measurements. The motor and cognitive development was assessed through the following tests: Movement Assessment Battery for Children (M-ABC) and Wechsler Preschool and Primary Scale of Intelligence – Third Edition; WPPSI I–III). A hearing test (tone audiometry), visual test and lung function testing (spirometry) were also performed.

### Definitions

***Probiotic use*** was defined as prophylactic administration of *Lactobacillus acidophilus*/*Bifidobacterium infantis* to VLBWI. Probiotic use was as follows: 41/48 centers used probiotics prophylactically. 38/41 centers administered probiotics to all VLBWI, 3 centers restricted probiotic use to infants with a birth weight <1000 g. All centers started probiotics (1 capsule/day) on day 1–3 of life and continued treatment for 28 days or until full enteral feeds (150 ml/kg) were reached.

***Antenatal exposure to antibiotic therapy*** was defined as antenatal antibiotic treatment of mothers of VLBWI (percentage of neonates whose mothers got any dose of antibiotics within 5 days before birth)).

***Postnatal exposure to antibiotic therapy*** was defined as antibiotic treatment of VLBWI (percentage of neonates who got any direct dose of antibiotics after birth; denomination: number of infants enrolled in GNN who were discharged home). Indirect exposure (for example by human milk) was not considered.

***Growth velocity*** was defined of growth (head circumference or body length) in mm/day (difference of the parameter at birth and at discharge/number of days in hospital).

### Statistical analysis

Data analysis was performed using the SPSS 22.0 data analysis package (Munich, Germany). Hypotheses were evaluated with two-sided tests including Fisher’s exact test and Mann-Whitney U test. A *p* value < 0.05 was considered as statistically significant for two-sided tests, Bonferroni corrections were made for multiple comparisons. Z-scores were calculated for birth weight, length and head circumference according to Voigt *et al*.^31^ and for anthropometric parameters according to Kromeyer-Hauschild *et al*.^32^ To determine potential associations between administration of probiotics and growth rates we conducted linear regression analyses with known confounding variables, i.e. gestational age per week, birth weight in 100 g steps, gender, multiple birth and maternal descent. Effect size and 95% confidence intervals (CI) were calculated. A p-value of <0.05 was considered statistically significant. For primary and subgroup analyses, we used a uniform dataset with available data for all metric parameters. Missing data were not included.

### Ethics

The study including all experimental protocols was approved by the local committee on research in human subjects of the University of Lübeck (08–022; 03.12.2010) and the local ethical committees at the other study centers. Informed consent was obtained from all subjects. All methods were carried out in accordance with relevant guidelines and regulations, specifically: the Declaration of Helsinki, the current revision of ICH Topic E6, the Guidelines for Good Clinical Practice, and the Guidelines of the Council for International Organization of Medical Sciences, the WHO (“Proposed International Guidelines For Biomedical Research Involving Human Subjects”).

## Electronic supplementary material

Supplementary Information
