# Supplementary material for: Lactobacillus acidophilus/Bifidobacterium infantis probiotics are associated with increased growth of VLBWI among those exposed to antibiotics
Source: Sci Rep. 2017 Jul 17;7:5633. doi: 10.1038/s41598-017-06161-8 (PMC5514087; doi:10.1038/s41598-017-06161-8)
Supplement: Supplementary file 1 — Supplementary Information [file 41598_2017_6161_MOESM1_ESM.pdf]

Supplemental information

Title Page

***Lactobacillus acidophilus / Bifidobacterium infantis* probiotics are associated with increased growth of VLBWI among those exposed to antibiotics**

Christoph Härtel[1] MD, Julia Pagel[1] MD, Juliane Spiegler, MD[1], Janne Buma[1], Philipp Henneke[2] MD, PhD, Michael Zemlin[3] MD, Dorothee Viemann[4] MD, Christian Gille[5] MD, Stephan Gehring[6] MD, David Frommhold[7] MD, Jan Rupp[8] MD, Egbert Herting[1] MD, PhD and Wolfgang Göpel[1] MD

[1]Department of Pediatrics, University of Lübeck, Germany
[2]Center for Pediatrics and Adolescent Medicine and Center for Chronic Immunodeficiency, University Medical Center, Freiburg, Germany
[3]Department of Pediatrics, University of Marburg, Germany
[4]Department of Neonatology, Hanover Medical School, Germany
[5]Department of Neonatology University of Tübingen, Germany
[6]Department of Infectious Diseases, Gastroenterology and Pediatric Intensive Care, University of Mainz, Germany, MD
[7]Department of Neonatology University of Heidelberg, Germany
[8]Department of Infectious Diseases and Microbiology, University of Lübeck, Germany

Corresponding author:
Christoph Härtel, MD
Department of Paediatrics, University of Lübeck,
Ratzeburger Allee 160, 23538 Lübeck, Germany
Tel.: + 49-451-500 2685, FAX: + 49-451-500 6222
E-mail: christoph.haertel@uksh.de

Keywords: very-low-birth-weight infants, probiotics, antibiotics, weight, growth,
Word count:

Supplements

Supplement 1: Parameters on clinical record files for infants enrolled in the German Neonatal Network

Birth data of the enrolled infant

initials, date of birth, inborn/outborn, gender, multiple birth, feto-fetal transfusion syndrome and therapy, causes of premature birth (premature labor, chorioamnionitis, gestosis, HELLP syndrome, conspicuous CTG, abruption of placenta, anhydramnion, premature rupture of membranes > 5 days/timepoint, onset before/with contractions, anhydramnios) mode of birth (spontaneous, elective section, emergency section), APGAR 5', APGAR 10', pH of umbilical artery, base excess of umbilical artery, birth weight, length, circumference of head, body temperature at birth, gestational age in weeks + days, congenital anomalies (life threatening, letal, CRIBS score)

Resuscitation and support within first 60 minutes of life:

Timepoint of resuscitation, sustained inflation, LISA (less invasive surfactant application), surfactant via endotracheal tube, intubation, bicarbonate infusion, application of volume, adrenalin, cardiopulmonary resuscitation, lactate levels within first 60 minutes, lowest mean arterial pressure within first 24 hours, use of inotropes within first 24 hours

Maternal data:

age, ethnicity, gravida, para, antenatal corticosteroids (type, completed cycle), tocolysis, cerclage, pessar, progesterone, admission to hospital > one week before birth, antenatal antibiotics (agents listed)

Individual therapy of the infant:

prophylactic drugs (vitamin K, teicoplanin/vancomycin, fluconazole, indomethacin, erythropoietin, others), antibiotic therapy with specification of given antibiotics (agents listed), specification of inotrope therapy (dopamine, noradrenaline, dobutamine, adrenaline), analgetics (acetaminophen, morphine, thiopental, pentobarbital, midazolam, fentanyl, phenobarbital, chloral hydrate, piritramid, sufentanil, propofol, diazepam, others), surfactant (including number of applications), mode of surfactant application, diuretics (furosemide, hydrochlorothiazide, spironolactone), inhalative medicaments (salbutamol, NO, budesonide, ipratropium

bromide, fluticasone, others), transfusions (amount of red blood cell, platelet and fresh frozen plasma transfusions), caffeine, theophylline, doxapram, other drugs: acetylcysteine, ambroxol, calcium gluconate, calcium glycerophosphate, calcium phosphate, dexamethasone, iron, glucose, hydrocortisone, ibuprofen, indomethacin, insulin, L-thyroxin, natrium gluconate, natrium glycerophosphate, bicarbonate, NaCl, omeprazole, phosphate, prednisolone, ranitidine, sildenafil, ursodeoxycholic acid, vitamins, zinc, others), probiotics (Lactobacillus acidophilus + Bifidobacteria infantis), vaccination (hexa polyvalent vaccines, pneumococcal vaccines, palivizumab, rotavirus vaccination), stopping of application of medicaments due to adverse events, used central venous lines (central venous catheter, umbilical cord catheter, including material of catheter, silicone, polyurethane) and possible complications, arterial catheter (umbilical artery catheter, peripheral artery catheter), complications of central access (infection, thrombosis), start of oral feeding, time to reach 150 ml per kg oral feeding in days, length of stay of intravenous catheter, type of oral nutrition during hospital stay and at discharge (breast milk, donated breast milk, special preterm milk, industrial milk, fortification of milk), begin and ending of oxygen supply, oxygen supply at discharge, CPAP (CPAP, CPAP with IMV, HFO-CPAP, highflow nasal cannula), type of ventilation (conventional ventilation [SIMV, IMV…], high frequency ventilation), time intervals of invasive ventilation, date of completed ventilation supply (inclusive CPAP), pulmonary interstitial emphysema, pneumothorax (during invasive ventilation, non-invasive ventilation, spontaneous breathing), drainage of pneumothorax, sepsis (pathogen) within/after 72 hours of admission, pneumonia within/after 72 hours of admission, necrotizing enterocolitis II/III, cranial ultrasound (grade of intracerebral haemorrhage I-IV, periventricular leukomalacia), hip dysplasia (grade Graf), retinopathy of prematurity (grade 0-5) therapy of retinopathy of prematurity (bevacizumab, laser, kryotherapy), result of screening for hearing loss, date and type of surgery (PDA, NEC, FIP, ventriculoperitoneal shunt, herniotomy, others), bronchopulmonary dysplasia, date of discharge, weight, length and head circumference at discharge, discharge home or to another facility, cause of death.

Supplemental table 1 Clinical characteristics of infants stratified to probiotics exposure

Parameter	Infants without probiotics (n=2305)	Infants with probiotics (n=6229)	p
**Cause of preterm birth#**			
Preterm labour	33.5	37.1	0.002
PPROM + Anhydramnios > 5d	4.5	5.6	0.1
Chorioamnionitis	19.9	22.2	0.02
Pre-eclampsia	8.8	7.8	0.1
HELLP syndrome	8.0	9.0	0.1
Pathological CTG	21.3	20.4	0.3
Pathological Doppler/IUGR	22.4	21.1	0.2
Placental abruption	7.4	7.7	0.6
**Gender, female**	48.8	48.9	0.9
**Multiple birth**	35.2	35.1	0.9
**Maternal background**			
Germany	74.7	71.4	<0.001
Europe/Russia	9.5	11.5	
Turkey/Middle East	6.7	7.7	
Asia	1.4	1.9	
Africa	4.6	5.6	
**Apgar 5 min**	8/8	8/8	0.8
**Apgar 10 min**	9/9	9/9	0.8
**Umbilical artery pH**	7.31/7.32	7.32/7.33	0.2
**Umbilical artery base excess**	-2.9/-2.2	-2.6/-2	0.01
**NEC surgery**	50/2303 (2.0)	109/6226 (1.8)	0.2<br>*OR 0.66 (0.47-0.93)<br>adj. p=0.017
**NEC or FIP surgery**	89/2303 (3.9)	230/6226 (3.7)	0.7 /<br>*OR 0.77 (0.59-0.99)<br>adj. p= 0.049

p-values were derived from two-sided Fisher´s exact test.
multiple causes of preterm birth were possible
*odds ratios (OR, 95% CI, adjusted p) were derived from logistic regression analysis including gestational age per week and prophylactic use of probiotics on the risk for surgical NEC and surgical NEC or FIP
PPROM prolonged premature rupture of membranes
HELLP highly elevated liver enzymes and low platelets
CTG cardiotocography, IUGR intrauterine growth restriction

Supplemental table 2 Antenatal antibiotics

**Antenatal parameter**	**% of infants exposed to antenatal antibiotics**
**Cause of preterm birth#**	
All (n=11929)	50.4
Preterm labour (n=4353, 36.4%)	70.2
PPROM (n=3587, 30.0%)	82.4
Chorioamnionitis (n=2629, 22.0%)	89.1
Pre-eclampsia (n=935, 7.8%)	18.6
HELLP syndrome (n=1021, 8.5%)	19.9
Pathological CTG (n=2459, 20.6%)	37.5
Pathological Doppler/IUGR (n=2542, 21.2%)	21.8
Placental abruption (n=588, 4.9%)	43.8
**Antibiotic classes**	
Penicillins	23.4
Cephalosporins	24.7
Metronidazol	7.0
Carbapenems	2.7
Makrolides	0.7
Others	8.4

The table describes the percentage of infants exposed to antibiotics within the last five days before birth.
multiple causes of preterm birth were possible
PPROM prolonged premature rupture of membranes
HELLP highly elevated liver enzymes and low platelets
CTG cardiotocography, IUGR intrauterine growth restriction

Supplemental table 3 Exposure to postnatal antibiotics (all GNN infants)

**Antibiotic classes**	**% of infants (n=11929) exposed**
**Penicillins, all**	68.0
Ampicillin / Ampicillin+Sulbactam	60.8 / 9.7
Piperacillin / Tazobactam+Piperacillin	20.2 / 6.9
Flucloxacillin	8.0
Mezlocillin	4.4
**Aminoglykosides, all**	63.2
Gentamycin	48.2
Tobramycin	15.8
Amikacin	8.3
**Glycopeptides, all**	35.4
Vancomycin	28.1
Teicoplanin	13.0
**Cephalosporins, all**	43.0
Cefotaxim	29.8
Ceftazidim	14.2
Cefuroxim	10.0
Cefazolin	1.9
**Carbapenems, all**	21.6
Meropenem	17.4
Imipenem	5.2
**Makrolides, all**	12.0
Erythromycin	7.8
Clarithromycin	5.1
**Others**	
Metronidazol	4.2
Fosfomycin	1.9
Linezolid	1.3
Clindamycin	0.3

The table describes the percentage of infants who had postnatal antibioticsdirectly administered throughout the primary stay in hospital. Classes of antibiotics and single antibiotics drugs are specified.
